# Hydrogen gas and olfactory dysfunction in Alzheimer’s disease: the Hydrogen–Olfactory–Limbic hypothesis

**DOI:** 10.3389/fnagi.2026.1937419

**Published:** 2026-10-02

**Authors:** Ligao Bao, Dunfu Eer

**Affiliations:** 1 Graduate School of Science, The University of Tokyo, Tokyo, Japan; 2 Department of Immunology, Graduate School of Medicine and Faculty of Medicine, The University of Tokyo, Tokyo, Japan

**Keywords:** Alzheimer’s disease, hydrogen gas, mitochondrial dysfunction, molecular hydrogen, neuroinflammation, olfactory bulb, olfactory dysfunction, olfactory epithelium

## Abstract

Olfactory dysfunction is increasingly recognized as an early clinical feature of Alzheimer’s disease (AD), often preceding measurable cognitive impairment and associated with subsequent cognitive decline. Despite this relatively consistent clinical association, the anatomical, cellular, and physiological substrates underlying olfactory dysfunction in AD remain poorly understood. Post-mortem human studies have identified amyloid-β (Aβ) and tau pathology in olfactory structures, including the olfactory epithelium and anterior olfactory nucleus. However, the available anatomical evidence remains limited, and the presence of these pathological proteins does not establish that they cause olfactory dysfunction. Likewise, it remains uncertain whether early olfactory impairment reflects peripheral sensory dysfunction, neuronal loss, synaptic or circuit abnormalities, altered higher-order olfactory processing, disruption of olfactory–limbic networks, or a combination of these processes. Although the olfactory epithelium possesses lifelong regenerative capacity, whether this capacity is altered during AD progression remains unresolved. Molecular hydrogen (H₂) has demonstrated antioxidant, anti-inflammatory, mitochondrial-modulatory, and cytoprotective effects in diverse experimental systems. However, direct evidence that hydrogen modifies AD-associated olfactory dysfunction is currently lacking. In this Perspective, we propose the Hydrogen–Olfactory–Limbic hypothesis as an experimentally testable framework rather than an established therapeutic mechanism. We suggest that molecular hydrogen can serve as an experimental intervention to determine whether modulating oxidative, inflammatory, and metabolic processes affects olfactory function and olfactory–limbic circuit physiology during AD progression. This framework integrates behavioral, anatomical, molecular, electrophysiological, regenerative, and connectivity measurements to address two fundamental questions: what causes early olfactory dysfunction in AD, and can molecular hydrogen modify biologically relevant processes associated with this phenotype?

## Graphical abstract

**Figure:**
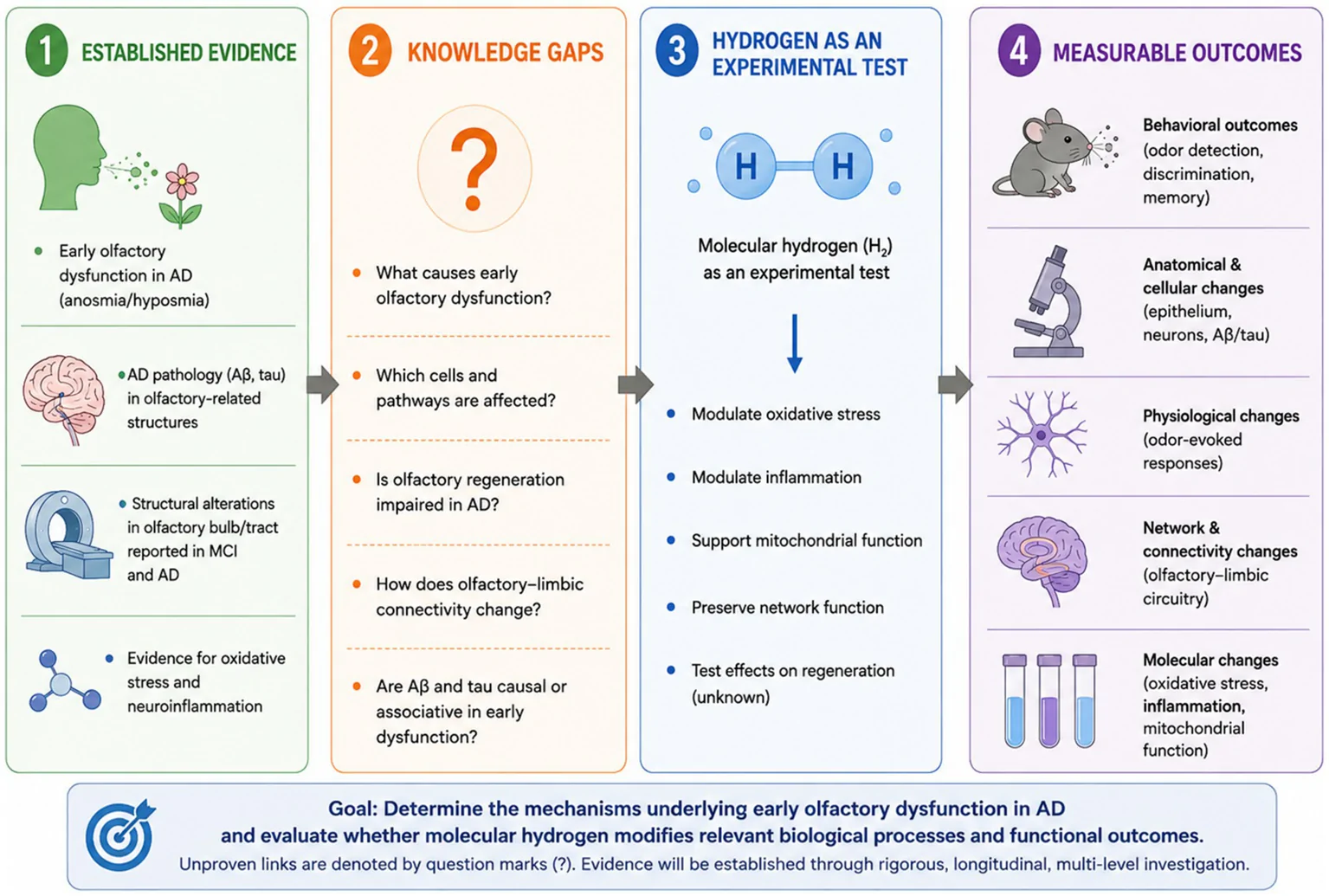
Hydrogen–Olfactory–Limbic hypothesis in Alzheimer’s disease: from established evidence to testable outcomes. The proposed framework distinguishes established observations from unresolved mechanisms underlying olfactory dysfunction in Alzheimer’s disease (AD). Clinical studies support early olfactory impairment, while primary human neuropathological studies demonstrate Aβ and tau pathology in peripheral and central olfactory structures, including the olfactory epithelium and the anterior olfactory nucleus. Structural alterations of the olfactory bulb and tract have also been reported. However, these observations do not establish the anatomical or physiological cause of olfactory dysfunction, and causal relationships among Aβ/tau pathology, neuronal dysfunction, olfactory epithelial regeneration, and impaired olfaction remain unresolved. Molecular hydrogen (H₂) is therefore presented as an experimental intervention rather than an established therapy. Its reported antioxidant, anti-inflammatory, and mitochondrial-modulatory actions provide candidate mechanisms that can be directly tested. Question marks denote unproven relationships. Proposed outcomes include olfactory behavior, epithelial and neuronal integrity, Aβ/tau pathology, regenerative activity, odor-evoked neuronal responses, olfactory–limbic connectivity, oxidative stress, inflammation, and mitochondrial function. Longitudinal integration of these measurements may clarify the mechanisms underlying early olfactory dysfunction and determine whether molecular hydrogen modifies biologically relevant processes or functional outcomes.

## 1 Introduction

Alzheimer’s disease (AD) is the most prevalent cause of dementia and poses a major medical and socioeconomic challenge worldwide. Extracellular amyloid plaques and intracellular neurofibrillary tangles are the defining neuropathological features of AD, but disease progression involves a broader range of interacting processes, including oxidative stress, mitochondrial dysfunction, chronic neuroinflammation, vascular abnormalities, synaptic impairment, and altered neural plasticity (Livingston et al., 2020; Swerdlow, 2018; Heneka et al., 2015; Leng and Edison, 2021; van Dyck et al., 2023).

The development of anti-amyloid therapies shows that modifying a major component of AD pathology can slow clinical progression in some patients. Nevertheless, the magnitude of clinical benefit remains limited (van Dyck et al., 2023), underscoring the need to identify additional biological processes and neural systems that may become dysfunctional early in the disease course.

The olfactory system is particularly relevant in this context. Impairments in odor identification, discrimination, detection, and memory have been reported in individuals with mild cognitive impairment (MCI) and AD (Doty, 2017; Murphy, 2019; Devanand et al., 2015; Roberts et al., 2016). Longitudinal studies further indicate that impaired olfactory performance is associated with subsequent cognitive decline and an increased risk of developing dementia (Devanand et al., 2015; Roberts et al., 2016). Thus, compared with many proposed mechanistic explanations for olfactory dysfunction, the clinical association between impaired olfaction and AD is relatively well established.

What is considerably less clear is why olfactory function becomes impaired. Primary neuropathological studies have demonstrated that AD-associated pathology can occur within components of the olfactory system. Aβ and tau abnormalities have been identified in the anterior olfactory nucleus and olfactory epithelium (Saiz-Sanchez et al., 2010; Arnold et al., 2010). However, the number of primary studies directly addressing the anatomical basis of olfactory dysfunction remains limited. Furthermore, detecting Aβ or tau in an olfactory structure demonstrates pathological association but does not establish that either protein is responsible for impaired smell.

An even greater knowledge gap concerns physiology. It remains unclear how AD alters odor-evoked neuronal activity, synaptic transmission, sensory coding, oscillatory activity, or communication among different components of the olfactory network. Consequently, early olfactory dysfunction could arise at multiple levels, including the olfactory epithelium, olfactory bulb, higher-order olfactory cortices, limbic structures involved in odor memory, or distributed interactions among these regions.

The olfactory system is also unusual because olfactory sensory neurons can be continuously replaced throughout adult life (Brann and Firestein, 2014; Mobley et al., 2014). This regenerative property raises important questions about neuronal maintenance in aging and neurodegenerative disease. However, lifelong olfactory neurogenesis should not be taken as evidence that this process is necessarily impaired in AD. Whether proliferation, differentiation, maturation, survival, axonal targeting, or functional integration of newly generated olfactory sensory neurons changes systematically during AD progression remains to be determined.

These unresolved questions are especially important when considering potential interventions. Molecular hydrogen (H₂) has attracted interest because experimental studies have demonstrated antioxidant, anti-inflammatory, mitochondrial-modulatory, and cytoprotective effects (Ohsawa et al., 2007; Ohta, 2014, 2015; Ichihara et al., 2015). Hydrogen can rapidly diffuse through biological membranes, and its biological actions have been examined in multiple experimental disease models (Ohsawa et al., 2007; Ohta, 2014, 2015; Ichihara et al., 2015).

However, evidence that molecular hydrogen directly improves AD-associated olfactory dysfunction is lacking. Its effects on olfactory sensory neurons, olfactory epithelial homeostasis, olfactory bulb circuitry, odor-evoked physiology, and olfactory–limbic connectivity in AD remain unknown.

Accordingly, this Perspective does not assume that neuronal degeneration or regenerative failure causes olfactory dysfunction, nor that hydrogen necessarily prevents or reverses these processes. Instead, we propose the Hydrogen–Olfactory–Limbic hypothesis as a framework for generating experimentally testable questions. Specifically, we ask whether molecular hydrogen can modify biological processes associated with AD and whether such modification produces measurable changes in olfactory function and in olfactory–limbic network physiology.

## 2 Olfactory dysfunction as an early clinical feature of Alzheimer’s disease

Among the observations linking the olfactory system to AD, clinical evidence is the most consistent. Olfactory dysfunction can be detected in individuals with MCI and AD, and impaired odor identification has been associated with subsequent cognitive deterioration (Doty, 2017; Murphy, 2019; Devanand et al., 2015; Roberts et al., 2016).

Devanand et al. (2015) demonstrated that olfactory deficits predicted cognitive decline and Alzheimer’s dementia in an urban community. Roberts et al. (2016) similarly reported an association between olfactory dysfunction, amnestic MCI, and AD dementia. Together with broader analyses of sensory impairment in AD (Doty, 2017; Murphy, 2019), these studies support the potential value of olfactory testing as a relatively inexpensive and non-invasive component of early clinical assessment.

Nevertheless, an association between olfactory impairment and future cognitive decline does not establish the biological mechanism responsible for that impairment. Olfactory performance depends on multiple levels of neural processing. Odorants are initially detected by olfactory sensory neurons in the olfactory epithelium. Their axons project to glomeruli in the olfactory bulb, where sensory information is transformed by complex neuronal circuits before being transmitted to the piriform cortex, amygdala, entorhinal cortex, and other regions associated with perception, emotion, learning, and memory (Shepherd, 2004).

An abnormal odor-identification test result could therefore arise from peripheral sensory dysfunction, altered olfactory bulb processing, impaired higher-order odor representation, memory dysfunction, or combinations of these abnormalities. This distinction is especially important in AD because memory, attention, and semantic processing can themselves influence performance on commonly used olfactory tests.

Furthermore, olfactory impairment is not specific to AD. Age-related changes, other neurodegenerative diseases, sinonasal disorders, environmental exposures, and numerous other conditions can affect smell (Doty, 2017; Murphy, 2019; Kovács, 2004). Consequently, olfactory testing should not be interpreted as an AD-specific diagnostic tool when used in isolation.

Thus, although early olfactory dysfunction is well supported clinically, its biological interpretation warrants considerable caution. The major challenge is no longer simply to demonstrate that smell is impaired in AD but to identify the anatomical and physiological substrates underlying that impairment.

## 3 What do we know about olfactory pathology in AD?

Neuropathological observations provide direct evidence that AD-associated pathology can involve components of the olfactory system. However, the amount of primary anatomical evidence is considerably smaller than might be assumed from the extensive clinical literature on olfactory dysfunction.

Saiz-Sanchez et al. (2010) examined the anterior olfactory nucleus in human AD tissue and demonstrated abnormalities involving tau and Aβ within this olfactory-associated structure. This study provides primary anatomical evidence that classical AD-related pathology can occur in a central component of the olfactory system.

Importantly, primary evidence is not restricted to central olfactory structures. Arnold et al. (2010) examined post-mortem olfactory epithelium from a large series of subjects and identified Aβ and paired helical filament-tau pathology in neuropathologically confirmed AD cases. Their findings provide direct anatomical evidence that peripheral olfactory tissue can also exhibit AD-associated pathological changes.

Together, these studies demonstrate that both peripheral and central components of the olfactory system can contain AD-associated pathology (Saiz-Sanchez et al., 2010; Arnold et al., 2010). Nevertheless, their interpretation requires caution.

First, the presence of Aβ or tau does not establish that these proteins caused anosmia or hyposmia during life. Post-mortem anatomical studies can demonstrate pathological localization and association with disease, but establishing a causal relationship with olfactory dysfunction requires correspondence among pathological burden, cellular or circuit abnormalities, and objectively measured olfactory performance.

Second, the components of the olfactory pathway may not be affected uniformly. Pathological changes in the olfactory epithelium could alter peripheral sensory input, whereas abnormalities in the olfactory bulb or anterior olfactory nucleus could affect central processing. Pathology in the piriform or entorhinal cortex could influence odor perception, identification, or memory even if peripheral sensory neurons remain functional.

Third, much of the literature on olfactory pathology in AD consists of reviews that synthesize a relatively limited primary evidence base. For example, Attems and colleagues provide an important overview of olfaction and aging (Attems et al., 2015), but such reviews should not be interpreted as independent primary anatomical evidence. Recognizing this distinction highlights an important deficiency in the field: despite extensive clinical documentation of olfactory impairment, surprisingly few studies have directly linked specific anatomical abnormalities to functional olfactory deficits in AD. Neuroimaging provides complementary evidence of structural involvement. MRI-derived atrophy of the olfactory bulb and tract has been reported in individuals with MCI and AD (Thomann et al., 2009). However, structural alterations alone cannot determine whether they cause olfactory impairment, develop secondary to altered sensory input, or arise in parallel as part of a broader neurodegenerative process.

Therefore, current evidence supports an association between AD and pathological or structural abnormalities within the olfactory system, but the causal anatomical substrate of olfactory dysfunction remains unresolved. This distinction underpins the central argument of this Perspective: identifying pathology within an olfactory structure is not equivalent to explaining olfactory dysfunction.

## 4 The major missing link: olfactory physiology

If the anatomical evidence is limited, the physiological evidence is even more incomplete. Relatively little is known about how AD pathology alters odor-evoked activity in defined olfactory neuronal populations or circuits.

Several fundamental questions remain unanswered. Do olfactory sensory neurons exhibit altered odor responsiveness in early AD? Are glomerular sensory representations disrupted? Does mitral or tufted cell output change? Are inhibitory circuits within the olfactory bulb altered? Does the temporal organization of olfactory activity, including oscillatory dynamics, become abnormal?

Similarly, it remains unclear whether communication between the olfactory bulb and higher-order olfactory or limbic regions changes before cognitive impairment becomes clinically apparent.

These questions are important because the olfactory system has extensive anatomical connections with structures involved in emotion and memory (Shepherd, 2004). Nevertheless, anatomical connectivity should not be taken as evidence that AD pathology necessarily propagates from the olfactory bulb to the hippocampus or that diminished olfactory input causes cognitive deterioration.

A major objective for future research should therefore be to integrate olfactory behavioral testing with direct physiological measurements. Electrophysiology, calcium imaging, functional imaging, and circuit-specific approaches could determine whether behavioral dysfunction corresponds to identifiable abnormalities in sensory coding, synaptic transmission, or network activity.

Longitudinal studies would be particularly informative because they could determine whether physiological dysfunction precedes, accompanies, or follows the appearance of AD-associated pathology.

For example, if odor-evoked neuronal abnormalities appear before substantial Aβ or tau accumulation, this would argue against a simple model in which protein deposition directly causes olfactory dysfunction. Conversely, a strong temporal relationship between pathology, circuit dysfunction, and olfactory behavior would provide a basis for subsequent causal experiments.

This distinction is central to determining whether olfactory dysfunction is primarily a biomarker of broader disease, a consequence of local pathology, or a functional component of disease-associated network disruption.

## 5 Olfactory regeneration in AD: an unresolved question

The olfactory epithelium is unusual among adult neural tissues because it retains substantial regenerative capacity throughout life. Basal stem and progenitor cells continuously generate new olfactory sensory neurons that differentiate, mature, and extend axons toward the olfactory bulb, thereby contributing to the maintenance and repair of the sensory epithelium (Brann and Firestein, 2014; Mobley et al., 2014). Adult neurogenesis has also been investigated in the broader context of neurodegenerative diseases (Winner and Winkler, 2015). However, findings from other neurogenic regions should not be extrapolated directly to the olfactory epithelium in AD.

Importantly, there is currently insufficient direct evidence that AD progressively impairs olfactory epithelial regeneration. It therefore remains unknown whether alterations in progenitor-cell proliferation, neuronal differentiation, maturation, survival, axonal targeting, or functional integration contribute to AD-associated olfactory dysfunction. Even if abnormalities in neuronal turnover are identified, their causal relationship to impaired olfaction would require direct experimental demonstration. Thus, regenerative failure should not presently be considered an established mechanism of early olfactory dysfunction in AD.

This uncertainty represents an important knowledge gap. Longitudinal studies should quantify basal-cell proliferation, lineage progression, newly generated olfactory sensory neurons, neuronal survival, and circuit integration at defined stages of AD pathology and relate these measurements to odor detection, discrimination, and memory.

Within the proposed Hydrogen–Olfactory–Limbic hypothesis, molecular hydrogen should therefore not be assumed to restore impaired neuroregeneration. Instead, hydrogen can serve as an experimental intervention to determine whether modulating oxidative, inflammatory, or metabolic processes affects olfactory epithelial homeostasis. Only if changes in regenerative indices are associated with functional improvement and subsequently shown to be mechanistically linked should olfactory regeneration be considered a component of hydrogen’s potential effects in AD.

## 6 Molecular hydrogen: what is empirically established?

Molecular hydrogen has been investigated as a biologically active gas across a range of experimental conditions. Ohsawa and colleagues reported that hydrogen reduced cytotoxic oxygen radicals and protected cells against oxidative injury (Ohsawa et al., 2007). Subsequent studies and reviews have described antioxidant, anti-inflammatory, anti-apoptotic, and metabolic effects of molecular hydrogen (Ohta, 2014, 2015; Ichihara et al., 2015).

Experimental studies provide evidence that hydrogen can influence oxidative and inflammatory processes (Xie et al., 2012; Huang et al., 2010; Yin et al., 2022). Hydrogen-rich saline has also been reported to improve memory function and reduce oxidative stress in an Aβ-induced rat model (Li et al., 2010). Nishimaki et al. (2018) examined molecular hydrogen in an animal model and in a randomized clinical study involving individuals with MCI.

These observations provide a rationale for investigating hydrogen in neurological disease, but they should not be interpreted as evidence that hydrogen protects the olfactory system in AD.

Many mechanistic studies of hydrogen have been conducted in non-olfactory tissues or in disease models unrelated to AD. For example, experimental evidence for anti-inflammatory actions includes studies of lipopolysaccharide-induced injury (Xie et al., 2012; Yin et al., 2022). These studies demonstrate biological activity but provide only indirect support for an olfactory application.

Similarly, hydrogen-associated activation of pathways such as Nrf2 has been demonstrated in experimental contexts outside the AD olfactory system (Fang et al., 2020; Liu et al., 2019). These findings establish mechanistic possibilities rather than demonstrated olfactory mechanisms.

The appropriate question is therefore not whether hydrogen is an olfactory neuroprotective or neuroregenerative therapy. Instead, the question is whether its experimentally demonstrated biological actions can modify processes that are functionally relevant to AD-associated olfactory dysfunction.

## 7 Potential effects of hydrogen on mechanisms relevant to olfactory dysfunction

Several biological actions of hydrogen warrant experimental investigation in the olfactory system, particularly oxidative stress, neuroinflammation, and mitochondrial dysfunction. However, these mechanisms should be regarded as candidate pathways rather than established causes of AD-associated olfactory impairment.

### 7.1 Oxidative stress

The olfactory epithelium occupies a distinctive interface between the nervous system and the external environment. Olfactory sensory neurons are repeatedly exposed to airborne chemicals, pollutants, pathogens, and oxidants (Kovács, 2004). Oxidative mechanisms may therefore influence olfactory tissue homeostasis.

Hydrogen has demonstrated antioxidant effects in multiple experimental systems (Ohsawa et al., 2007; Ohta, 2014, 2015; Ichihara et al., 2015). However, whether oxidative stress contributes directly to early olfactory dysfunction in AD remains uncertain.

Future studies should therefore measure oxidative markers and olfactory function simultaneously. If hydrogen reduces oxidative stress without altering olfactory performance, oxidative abnormalities may not be sufficient to explain the functional deficit. Conversely, parallel biochemical and functional improvement would support—but not by itself prove—a mechanistic relationship that requires further experimental testing.

### 7.2 Neuroinflammation

Neuroinflammation is an important component of AD biology (Heneka et al., 2015; Leng and Edison, 2021). Molecular hydrogen can modulate inflammatory responses in experimental models, including reductions in pro-inflammatory signaling (Xie et al., 2012; Yin et al., 2022).

However, whether inflammatory activity within the olfactory epithelium or olfactory bulb is a major determinant of early AD-associated smell dysfunction remains unknown.

Future studies should quantify microglial and astrocytic states in olfactory structures while simultaneously assessing odor-guided behavior and neuronal physiology. Manipulating inflammatory pathways independently of hydrogen would then be necessary to determine whether inflammation is causally related to olfactory dysfunction.

### 7.3 Mitochondrial function

Mitochondrial dysfunction is strongly implicated in AD (Swerdlow, 2018; Reddy and Beal, 2008). Hydrogen has been reported to influence mitochondrial and oxidative processes in experimental systems (Ichihara et al., 2015; Xie et al., 2012).

The relevance of these effects to olfactory dysfunction remains speculative. Direct measurements of mitochondrial respiration, membrane potential, reactive oxygen species, and metabolic state in defined olfactory cell populations could determine whether mitochondrial abnormalities accompany functional impairment.

Hydrogen could then serve as an experimental perturbation to determine whether modifying these abnormalities alters olfactory physiology.

Thus, oxidative, inflammatory, and mitochondrial mechanisms should be treated as testable candidate pathways rather than assumed explanations for olfactory dysfunction.

## 8 The Hydrogen–Olfactory–Limbic hypothesis

Based on the empirical evidence and knowledge gaps described above, we propose the Hydrogen–Olfactory–Limbic hypothesis.

Its empirical foundation is intentionally limited to observations supported by existing evidence: olfactory dysfunction can occur early in the clinical course of AD; pathological and structural abnormalities have been identified in peripheral and central components of the olfactory system (Thomann et al., 2009; Saiz-Sanchez et al., 2010; Arnold et al., 2010); and molecular hydrogen has been shown to affect oxidative, inflammatory, and metabolic processes (Ohsawa et al., 2007; Ohta, 2014, 2015; Ichihara et al., 2015; Fang et al., 2020; Xie et al., 2012; Huang et al., 2010; Nishimaki et al., 2018; Yin et al., 2022; Li et al., 2010; Liu et al., 2019).

What connects these observations remains unknown.

We hypothesize that one or more cellular or circuit-level abnormalities within the olfactory system contribute to measurable olfactory dysfunction during AD progression. These abnormalities could involve peripheral sensory neurons, olfactory bulb circuits, higher-order olfactory regions, or functional interactions between olfactory and limbic structures.

Within this framework, hydrogen serves as an experimental perturbation rather than an established therapy.

If hydrogen alters oxidative, inflammatory, or metabolic processes and this alteration is accompanied by improved olfactory function, the next question is whether the relationship is causal. Conversely, if molecular changes occur without functional improvement, this would argue against those processes being sufficient determinants of olfactory dysfunction.

The “limbic” component of the hypothesis likewise requires direct testing. The olfactory system has anatomical connections with regions involved in memory and emotion (Shepherd, 2004), but the functional consequences of AD-associated olfactory dysfunction for these networks remain uncertain.

Accordingly, the proposed Hydrogen–Olfactory–Limbic framework should not be interpreted as a demonstrated pathway of disease propagation from the olfactory system to the hippocampus. Instead, it defines an experimentally accessible network in which sensory function, pathology, cellular physiology, molecular mechanisms, and functional connectivity can be investigated longitudinally.

## 9 Experimental tests of the hypothesis

A rigorous evaluation of the Hydrogen–Olfactory–Limbic hypothesis should begin by establishing the mechanisms of olfactory dysfunction independently of hydrogen treatment.

First, longitudinal olfactory phenotyping should be performed in appropriate AD models before and during the development of cognitive abnormalities. Behavioral assays should assess odor detection threshold, discrimination, habituation/dishabituation, associative odor learning, and odor memory.

Second, anatomical analyses should determine whether functional impairment corresponds to measurable abnormalities in the olfactory epithelium, olfactory bulb, anterior olfactory nucleus, piriform cortex, or other relevant structures. Aβ and tau should be quantified, but their presence should not be assumed to explain dysfunction.

Third, neuronal physiology should be examined directly. Odor-evoked responses in olfactory sensory neurons, glomerular activity, mitral/tufted cell output, local inhibitory circuitry, and oscillatory dynamics could reveal functional abnormalities not apparent from conventional histological analysis.

Fourth, olfactory epithelial regeneration should be measured rather than presumed impaired. Proliferation and lineage-tracing approaches, combined with markers of progenitor cells and immature and mature olfactory sensory neurons, could establish whether neuronal turnover changes with disease stage.

Only after these baseline relationships have been established should the effects of molecular hydrogen be assessed.

Hydrogen-treated and control animals should undergo equivalent behavioral, anatomical, physiological, inflammatory, metabolic, and regenerative analyses. Such an approach would determine not only whether hydrogen alters olfactory function, but also which biological changes accompany any functional effect.

Experiments that independently manipulate candidate pathways would then be required to establish causality. For example, if hydrogen simultaneously reduces inflammation and improves olfactory behavior, selective manipulation of inflammatory signaling would be required to determine whether the anti-inflammatory effect mediates the behavioral improvement.

Finally, olfactory–limbic connectivity should be evaluated using electrophysiology, functional imaging, neuronal tracing, or circuit-specific approaches. These experiments could determine whether early olfactory dysfunction is associated with altered functional communication between olfactory structures and memory-related networks.

Such an integrated strategy would convert the proposed framework from a conceptual model into a series of falsifiable experimental hypotheses.

## 10 Clinical translation: evidence before intervention

Clinical translation should follow mechanistic validation rather than precede it.

The association between impaired olfaction and subsequent cognitive decline supports ongoing evaluation of olfactory testing as part of multimodal risk assessment (Devanand et al., 2015; Roberts et al., 2016). However, olfactory dysfunction is not specific to AD and can occur with normal aging, other neurological diseases, and numerous sinonasal conditions (Doty, 2017; Murphy, 2019; Kovács, 2004).

Consequently, olfactory testing should ideally be combined with established clinical, cognitive, imaging, and molecular biomarkers rather than used as an isolated diagnostic measure.

Human studies of molecular hydrogen provide preliminary evidence that hydrogen administration is feasible and warrants further investigation (Nishimaki et al., 2018), but current evidence is insufficient to conclude that hydrogen can prevent or reverse AD-associated olfactory dysfunction.

An initial translational objective should therefore be modest: determine whether hydrogen administration produces measurable changes in olfactory performance and relevant biological markers in carefully characterized populations.

Such studies should ideally distinguish odor detection from higher-order odor identification and memory, because improvement in one component does not necessarily indicate restoration of the entire olfactory system.

If effects are detected, subsequent studies could determine whether changes in olfactory function correspond to changes in cognition, brain-network activity, inflammatory or oxidative biomarkers, or established AD biomarkers.

This staged approach avoids assuming that olfactory dysfunction is a causal driver of cognitive decline while preserving its potential value as an early and experimentally measurable phenotype.

## 11 Conclusion

Olfactory dysfunction is one of the most intriguing early clinical features of Alzheimer’s disease, yet its biological basis remains poorly understood. Clinical studies show that impaired olfactory performance can precede or predict cognitive deterioration, but the anatomical and physiological substrates underlying this relationship remain uncertain (Doty, 2017; Murphy, 2019; Devanand et al., 2015; Roberts et al., 2016; Attems et al., 2015).

Importantly, primary human neuropathological studies show that AD-associated Aβ and tau pathology can occur in both peripheral and central olfactory structures, including the olfactory epithelium and anterior olfactory nucleus (Saiz-Sanchez et al., 2010; Arnold et al., 2010). These observations strengthen the anatomical association between the olfactory system and AD but do not establish that such pathology causes olfactory dysfunction.

It remains uncertain whether early smell impairment arises from peripheral sensory dysfunction, neuronal loss, synaptic or circuit abnormalities, altered higher-order processing, disrupted olfactory–limbic communication, or combinations of these mechanisms. Similarly, although the olfactory epithelium has substantial regenerative capacity, there is currently insufficient evidence that this capacity is progressively impaired in AD.

Molecular hydrogen has demonstrated antioxidant, anti-inflammatory, metabolic, mitochondrial-modulatory, and cytoprotective effects in experimental systems (Ohsawa et al., 2007; Ohta, 2014, 2015; Ichihara et al., 2015; Fang et al., 2020; Xie et al., 2012; Huang et al., 2010; Nishimaki et al., 2018; Yin et al., 2022; Li et al., 2010; Liu et al., 2019), but its effects on AD-associated olfactory dysfunction remain unknown.

We therefore propose the Hydrogen–Olfactory–Limbic hypothesis not as an established neuroprotective or neuroregenerative pathway, but as an experimentally testable framework that connects these currently separate observations.

The immediate priority is to determine the anatomical, cellular, and physiological basis of early olfactory dysfunction. Only after these substrates have been defined can molecular hydrogen be rigorously evaluated for its ability to modify biologically relevant processes and functional outcomes.

In this formulation, the principal value of the olfactory system is not that it is assumed to represent an initiating site of neurodegeneration or regenerative failure. Rather, it provides a clinically relevant and experimentally accessible neural system in which early functional changes associated with AD can be investigated.

Defining these mechanisms will be essential before the therapeutic significance of hydrogen—or any intervention directed toward olfactory dysfunction—can be established.

## Data Availability

The original contributions presented in the study are included in the article/supplementary material, further inquiries can be directed to the corresponding author.
